# Daily high-frequency transcranial random noise stimulation of bilateral temporal cortex in chronic tinnitus – a pilot study

**DOI:** 10.1038/s41598-019-48686-0

**Published:** 2019-08-22

**Authors:** Peter M. Kreuzer, Timm B. Poeppl, Rainer Rupprecht, Veronika Vielsmeier, Astrid Lehner, Berthold Langguth, Martin Schecklmann

**Affiliations:** 1 Department of Psychiatry and Psychotherapy, University of Regensburg, Regensburg, Germany; 2 Department of Otorhinolaryngology, University of Regensburg, Regensburg, Germany; 3 Interdisciplinary Tinnitus Center of the University of Regensburg, Regensburg, Germany

**Keywords:** Health care, Neurology

## Abstract

Several studies emphasized the potential of single and multiple transcranial random noise stimulation (tRNS) sessions to interfere with auditory cortical activity and to reduce tinnitus loudness. It was the objective of the present study to evaluate the use of high-frequency (hf) tRNS in a one-arm pilot study in patients with chronic tinnitus. Therefore, 30 patients received 10 sessions of high frequency tRNS (100-640 Hz; 2 mA; 20 minutes) over the bilateral temporal cortex. All patients had received rTMS treatment for their tinnitus at least 3 months before tRNS. Primary outcome was treatment response (tinnitus questionnaire reduction of ≥5 points). The trial was registered at clinicaltrials.gov (NCT01965028). Eight patients (27%) responded to tRNS. Exactly the same number of patients had responded before to rTMS, but there were only two “double responders” for both treatments. None of the secondary outcomes (tinnitus numeric rating scales, depressivity, and quality of life) was significant when results were corrected for multiple comparisons. tRNS treatment was accompanied by tolerable side effects but resulted in temporal increases in tinnitus loudness in 20% of the cases (2 drop-outs). Our trial showed that hf-tRNS is feasible for daily treatment in chronic tinnitus. However, summarizing low treatment response, increase of tinnitus loudness in 20% of patients and missing of any significant secondary outcome, the use of hf-tRNS as a general treatment for chronic tinnitus cannot be recommended at this stage. Differences in treatment responders between tRNS and rTMS highlight the need for individualized treatment procedures.

## Introduction

Transcranial random noise stimulation (tRNS) is a non-invasive brain stimulation method using varying alternating currents to interfere with oscillatory brain activity^1^. Technically, tRNS represents a special form of transcranial alternating current stimulation (tACS) with the current alternating at random normally distributed frequencies. tRNS is typically applied as low frequency tRNS (lf-tRNS; frequency range 0,1–100 Hz), high frequency tRNS (hf-tRNS; frequency range 101–650 Hz) or as whole frequency tRNS (wf-tRNS; frequency range 0,1–650 Hz). tRNS has demonstrated more pronounced effects on motor cortex excitability than other non-invasive brain stimulation techniques such as anodal transcranial direct current stimulation (tDCS) or intermittent theta burst TMS (transcranial magnetic stimulation) (iTBS)^2^. Even short-term tRNS of 4 minutes was shown to modulate the blood oxygen level dependent (BOLD) signal in the human motor cortex^3^. Its putative mechanism of action is the enhancement of neuronal noise and the decrease of hypersynchronicity^4^. It was hypothesized that the main mechanism of tRNS action was based on repeated subthreshold stimulations, which might prevent homeostasis of the system and potentiate task-related neural activity^5^.

tRNS has been proposed as a treatment option for major depression^6^, schizophrenia^7^, neuropathic pain^8^, and fibromyalgia^9^. It has been shown to modulate pain perception in multiple sclerosis^10^. hf- tRNS has also been found to be able to enhance mathematical ability speed^11^ and perceptual learning^5^. In a case report the successful treatment of a patient suffering from “Red Ear Syndrome” by high-frequency tRNS was registered^12^. The “Red Ear Syndrome” contains a symptom complex of auricular erythema, attack-like local pain and tinnitus perception^13^.

In patients with tinnitus, the effects of single sessions of tDCS, tACS and low-frequency tRNS of the auditory cortex were compared regarding tinnitus loudness and the tinnitus-related distress after stimulation of the auditory cortex. A direct comparison of three non-invasive stimulation methods applied to the bilateral auditory cortex in 111 tinnitus patients demonstrated that the transient suppressive effect on tinnitus loudness and tinnitus related distress was larger after tRNS compared to tDCS and tACS. Both tDCS and tACS induced small and non-significant effects on tinnitus perceptions, suggesting lf-tRNS as a superior brain stimulation method for tinnitus suppression^14^.

A recent proof-of-concept study provides evidence on an added value of bilateral temporal lf-tRNS treatment following bifrontal tDCS in 40 patients reporting chronic tinnitus^15^. The differential effects of high- and low-frequency tRNS targeting the auditory cortex were investigated by Joos *et al*.^16^ in 154 chronic tinnitus patients. A total of 119 patients was treated with lf-tRNS, 19 with hf-tRNS and 16 with wf-tRNS. The effects were evaluated applying numeric rating scales for loudness and distress for the states pre- and post-stimulation. This study showed a significant reduction in tinnitus loudness for both lf-tRNS and hf-tRNS as well as reduced tinnitus-related distress with lf-tRNS. However, hf-tRNS resulted in a more pronounced reduction of loudness and distress in pure tone tinnitus than in narrow band noise tinnitus^16^. This is in line with results derived from motor-cortex tRNS applications indicating a higher effectiveness for hf-tRNS^1^.

Moreover, a sham-controlled trial demonstrated that resting state and steady state activity of the auditory cortex were lowered by hf-tRNS in a sample of healthy controls^4^ indicating that previously described mechanisms of actions derived from motor-cortical areas could potentially be transferred to the auditory cortex. Van Doren *et al*. applied EEG measures to examine tRNS induced changes in resting state activity and auditory steady state responses (ASSRs). 1000 Hz carrier frequency tones with amplitudes modulated at 20 Hz and 40 Hz applied in randomized order were applied as stimuli. Fourteen healthy subjects took part in this placebo-controlled randomized design study; each of the participants received 20 min of tRNS applied over the auditory cortices with 2 mA. The study resulted in significant increases regarding the ASSR in response to 40 Hz frequency modulated tone and non-significant trends towards an increase in mean theta band power and variability of the theta band power for the resting state data. Although the authors interpreted their results of tRNS induced increased excitability of the auditory cortex in line with tRNS effects on motor cortex excitability, it should not be concealed that stimulation parameters used in this study significantly differ from the “conventional” study protocols targeting motor cortical areas with regard to higher stimulation intensities and longer stimulation periods^4^.

In sum, single studies highlight the capability of different types of tRNS among tES techniques to modulate tinnitus loudness and distress effectively. Here we aimed to investigate the effects of multiple sessions of hf-tRNS in a one-arm pilot study in patients with chronic tinnitus who had already received repetitive transcranial magnetic stimulation.

## Methods

We included 30 patients with chronic tinnitus (age: 49.2 ± 10.9 years; sex: 4 females; mean hearing loss averaged over both sides and the range of the standard audiogram (250 Hz–8 kHz): 20.1 ± 12.5 dB HL; tinnitus distress: 45.8 ± 19.3 [range: 9–80] tinnitus questionnaire (TQ^17^; total score); tinnitus laterality: one purely right-sided, seven purely left-sided, 22 with tinnitus in both ears or within head; tinnitus duration: 96.0 ± 73.7 months).

Patients were stimulated with 2 mA (0 off-set) over the bilateral temporal cortex for 20 minutes (ramp times: 10 s; 5 cm × 7 cm electrodes). The cathode was always applied to the left hemisphere and the anode to the right hemisphere with the inferior center of the electrode over the EEG position T7/T8. The long side of the electrodes was oriented in anterior-posterior direction. We used hf-tRNS (100–640 Hz) applied by a NeuroConn (Ilmenau, Germany) DC-Stimulator Plus device for ten days. Stimulation procedures were similar to earlier published data in healthy controls^4^. Patients were treated for 10 consecutive working days beginning with Monday and pausing at weekend.

Study visits were done in the hospital, included measurement of tinnitus characteristics and questionnaires, and took place at baseline (treatment day 1), week2 (treatment day 10/end of treatment), follow-up visits were conducted in week4 and 12 (two and ten weeks after treatment, respectively).

All patients had previously received a rTMS treatment series 22.6 ± 27.1 months (range 3–108 months) before enrolment in the tRNS study. However, the main focus of the study was the investigation of tRNS in tinnitus in a sample of patients pre-treated with rTMS. However, it was not pre-specified that rTMS has to be standardized with respect to the rTMS protocol and the time difference between both treatments. The rationale was to be able to estimate differences in the efficacy of both treatments. Tinnitus distress for the patients was comparable between the baseline visit of the rTMS and the tRNS treatment (rTMS: 44.8 ± 17.6; tRNS: 46.6 ± 18.9; T = 1.073; df = 25; p = 0.293). Detailed rTMS treatment parameters differed between patients due to the participation in different rTMS studies in our center. We contrasted both types of treatment (tRNS vs. rTMS). For an overview of demographics and rTMS study parameters see supplementary information.

Treatment response (primary outcome) was defined as reduction in the tinnitus questionnaire (TQ; range 0–84)^17^ of five points or more according to Adamchic *et al*.^18^ from baseline to week 12. The Tinnitus Questionnaire (TQ) was initially developed by Hallam *et al*.^19^ and has been translated and adapted by Goebel and Hiller in 1994^17^. This instrument differentiates between emotional and cognitive distress, auditory perceptual difficulties and self-experienced intrusiveness produced by the tinnitus. Broadly accepted as an outcome measure in clinical studies on chronic tinnitus, the TQ can be employed both for comparative studies in different tinnitus-related institutions and for the evaluation of the relative effects of different treatment approaches.^17^

To investigate possible association of treatment response between tRNS and rTMS we calculated a Chi-Square-test. Secondary outcomes were changes in TQ, Major Depression Inventory (MDI) by Bech *et al*.^20^, numeric rating scales for tinnitus loudness, discomfort, annoyance, ignorability and unpleasantness (range 0–10 numeric analog scale), and WHO Quality of Life questionnaire (WHOQOL-BREF)^21^ over the course of the trial. Data were assessed according to international standards^22,23^ and registered in a tinnitus database following ICH-GCP-regulations^24^. Secondary outcome variables were used for direct contrast between baseline and final to see if tRNS has treatment effects. Analyses of variance with the dependent variables TQ, MDI, numeric ratings, and quality of life and with the independent factors time (visits) and treatment (tRNS, rTMS) were used to see if there is a difference of tRNS with respect to pre-treatment with rTMS. All results from statistical analyses of secondary outcomes were also calculated with corrections for multiple comparisons by using Bonferroni corrections. All data are displayed as mean ± standard deviation if not otherwise labelled. Significance threshold was set to 5%. We conducted a per-protocol analysis (n = 26). Missing data were replaced with last-observation-carried-forward method.

hf-tRNS is a relatively new type of brain stimulation in daily treatment. Therefore, further objectives were the assessment of adverse events and safety information by the application of standardized questionnaires at all visits. Data were available for 18 patients who answered on a 10-point numeric scale (1 (not) to 10 (extreme)) the occurrence of side effects. Cognitive effects were tested with measures of tonic and phasic alertness, working memory and divided attention as elicited by the Test of Attentional Performance (Psytest, Germany). Cognitive data were available for 23 patients.

Data were assessed according to international standards^25^ and registered in an established tinnitus database following ICH-GCP-regulations^22,23^. The trial was registered on clinicaltrials.gov (NCT01965028; date: 14/10/2013) and approved by the local ethics committee of the University Hospital Regensburg (13-101-0143). All participants gave written informed consent after a comprehensive explanation of the study procedures. All methods were performed in accordance with the relevant guidelines and regulations.

## Results

### Adverse events

**Figure 1:**
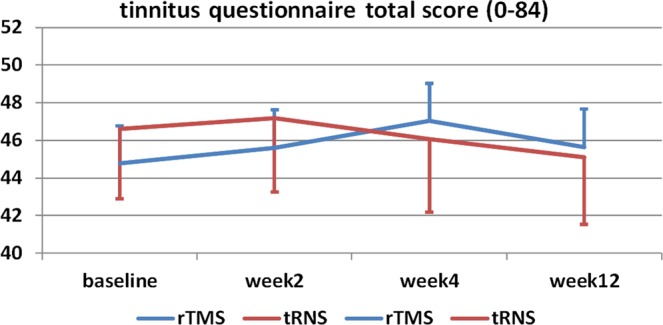
Tinnitus Questionnaire (TQ) total score over the course of the trial.

Six patients perceived temporal increases in tinnitus loudness (for two of them it was unbearable and they dropped out at treatment day 6 and 8; transient increases can occur during treatment thus the other patients did not quit) and two patients reported headaches (one drop-out). One patient did not finish the treatment due to personal reasons (overall four drop-outs). Reported adverse effects were the perception of mild to extreme pain (17% out of 18 patients), tingling (72%), burning (28%), tiredness (56%), nervousness (22%), concentration problems (39%), visual problems (11%), headaches (39%), itching (39%), dizziness (11%), lightnings (6%) and sounds (28%) during or after the stimulation. 44% reported sleeping problems. In general 17% rated the stimulation as unpleasant. One patient presented high ratings of all side effects. With respect to cognitive effects of the stimulation, quantitative analyses of norm T scores showed mean numeric increases over the course of the trial (Fig. 1). A detailed view on the single values indicates increases in performance of the majority of the patients, but also decreases in single parameters up to 7 out of 23 patients. Critical values are data below a T score of 30 which indicate norm-deviant values. Only 3 patients showed deviant values for single parameters after the stimulation. Most of them were already deviant or at borderline level at the beginning of the treatment. Another critical parameter would be decrease in performance by one standard deviation which was the case for 15 single values (out of 158). Compared with this 22 single values increased by at least one standard deviation.

### Primary outcome

Eight patients fulfilled the response criterion after tRNS treatment. There was a descriptive decrease in TQ from baseline to final visit for tRNS. In comparison, 8 patients had shown a response to previous rTMS treatment. But only two of them responded to both rTMS and tRNS. Chi-Square-test indicate no significant association (χ² = 0181; df = 1; p = 0.671). For detailed information with respect to kind of rTMS treatment and response to rTMS and tRNS see supplementary information. Most of the rTMS treatments included prefrontal and temporal/temporoparietal stimulation and indicated no specific association of treatment response and kind of treatment. This study was not designed as study to investigate the additive effects of rTMS treatment and tRNS treatment. rTMS treatment serves as control condition for statistical reasons but was not *a priori* planned as controlled study.

### Secondary outcomes

Contrasts from baseline to final visit for tRNS treatment resulted in significant decreases for tinnitus annoyance, unpleasantness and depressivity (Table 1). Analyses of variance with the dependent variables TQ, MDI, numeric ratings, and quality of life and with the independent factors time and treatment showed no significant effects of time (all F-values < 2.701; df = 3,75; all p-values > 0.052) or time by treatment (all F-values < 2.627; df = 3,75; all p-values > 0.056) except for the numeric rating “unpleasantness of tinnitus” (time by treatment: F = 4.294; df = 3,75; p = 0.008) and the quality of life domain “physical well-being” (time by treatment: F = 2.767; df = 3,69; p = 0.048). “Unpleasantness of tinnitus” decreased from baseline to week 2 (baseline vs. week 2 for tRNS: p = 0.002), but was also higher at baseline of the tRNS trial as compared to the rTMS treatment (tRNS vs. rTMS for baseline: p = 0.004;). Physical well-being decreased from week 2 to week 4 during the rTMS treatment (week 2 vs. week 4 for rTMS: p = 0.010). All observations for secondary outcome measures did not survive correction for multiple comparisons.

**Table 1:** Descriptive statistics of the dependent variables over the course of the trials.

	Treat-ment	Time				t-statistics (final vs. baseline for tRNS) df = 25	F-statistics
		Baseline begin of treatment	Week 2 end of treatment	Week 4 follow-up 1	Week 12 final visit		
tinnitus questionnaire (0–84)	tRNS	44.8 ± 17.6	45.6 ± 17.3	47.4 ± 18.3	45.7 ± 19.7	T = −0.952 p = 0.350 d = 0.187	time: F = 0.638; df = 3,75; p = 0.593 treatment: F = 0.106; df = 1,25; p = 0.747 time × treatment: F = 1.298; df = 3,75; p = 0.281
	rTMS	46.6 ± 18.9	47.2 ± 20.1	46.1 ± 19.9	45.1 ± 18.2		
tinnitus loudness rating (0–10)	tRNS	7.4 ± 1.6	6.9 ± 1.6	6.9 ± 1.8	7.0 ± 1.8	T = −1.122 p = 0.273 d = 0.220	time: F = 0.850; df = 3,75; p = 0.471 treatment: F = 0.118; df = 1,25; p = 0.734 time × treatment: F = 1.343; df = 3,75; p = 0.267
	rTMS	7.0 ± 1.8	6.9 ± 1.8	7.2 ± 1.9	6.9 ± 2.3		
tinnitus discomfort rating (0–10)	tRNS	7.8 ± 1.5	7.0 ± 1.9	7.2 ± 1.7	7.3 ± 1.7	T = −1.759 p = 0.091 d = 0.345	time: F = 2.701; df = 3,75; p = 0.052 treatment: F = 0.045; df = 1,25; p = 0.833 time × treatment: F = 1.243; df = 3,75; p = 0.300
	rTMS	7.4 ± 1.8	7.0 ± 1.8	7.4 ± 2.1	7.2 ± 2.4		
tinnitus annoyance rating (0–10)	tRNS	7.6 ± 1.6	6.9 ± 1.8	6.9 ± 2.0	6.9 ± 1.9	T = −2.095 p = 0.047 d = 0.411	time: F = 0.855; df = 3,75; p = 0.468 treatment: F = 0.607; df = 1,25; p = 0.443 time × treatment: F = 2.627; df = 3,75; p = 0.056
	rTMS	6.8 ± 2.1	6.8 ± 2.1	6.9 ± 2.5	7.0 ± 2.4		
tinnitus ignorability rating (0–10)	tRNS	7.6 ± 1.9	7.0 ± 2.2	7.0 ± 2.3	7.0 ± 2.5	T = −1.213 p = 0.237 d = 0.243	time: F = 1.010; df = 3,72; p = 0.393 treatment: F = 0.416; df = 1,24; p = 0.525 time × treatment: F = 0.370; df = 3,72; p = 0.775
	rTMS	7.1 ± 2.2	7.0 ± 2.1	7.0 ± 2.3	6.8 ± 2.7		
tinnitus unpleasantness rating (0–10)	tRNS	7.7 ± 1.6	6.7 ± 1.8	6.9 ± 1.8	7.0 ± 1.8	T = −2.360 p = 0.026 d = 0.463	time: F = 1.728; df = 3,75; p = 0.168 treatment: F = 0.260; df = 1,25; p = 0.615 time × treatment: F = 4.294; df = 3,75; p = 0.008
	rTMS	6.9 ± 2.1	6.9 ± 1.9	7.1 ± 2.2	7.1 ± 2.4		
quality of life physical health (4–20*)	tRNS	13.5 ± 3.3	13.7 ± 2.9	13.5 ± 3.1	14.1 ± 2.8	T = 1.748 p = 0.093 d = 0.343	time: F = 1.120; df = 3,69; p = 0.347 treatment: F = 0.004; df = 1,23; p = 0.949 time × treatment: F = 2.767; df = 3,69; p = 0.048
	rTMS	138 ± 3.1	14.0 ± 2.6	13.4 ± 2.3	13.5 ± 2.7		
quality of life psychological health domain (4–20*)	tRNS	13.3 ± 3.3	13.6 ± 2.9	13.8 ± 2.8	13.5 ± 2.8	T = 0.669 p = 0.509 d = 0.131	time: F = 0.373; df = 3,69; p = 0.772 treatment: F = 0.066; df = 1,23; p = 0.800 time × treatment: F = 1.651; df = 3,69; p = 0.186
	rTMS	13.6 ± 2.5	13.7 ± 2.5	13.2 ± 2.5	13.5 ± 2.8		
quality of life social relationships (4–20*)	tRNS	15.9 ± 2.5	15.6 ± 2.9	15.6 ± 2.7	15.5 ± 2.9	T = −0.781 p = 0.442 d = 0.153	time: F = 0.219; df = 3,69; p = 0.883 treatment: F = 3.483; df = 1,23; p = 0.075 time × treatment: F = 0.900; df = 3,69; p = 0.446
	rTMS	14.9 ± 3.1	15.2 ± 2.9	14.8 ± 3.0	15.0 ± 2.5		
quality of life environment (4–20*)	tRNS	16.0 ± 1.6	16.1 ± 1.7	16.3 ± 1.7	16.1 ± 1.8	T = 0.368 p = 0.716 d = 0.072	time: F = 0.007; df = 3,69; p = 0.999 treatment: F = 0.127; df = 1,23; p = 0.725 time × treatment: F = 1.072; df = 3,69; p = 0.367
	rTMS	16.2 ± 1.6	16.1 ± 1.7	15.9 ± 1.7	16.1 ± 1.7		
depressivity (0–50)	tRNS	9.1 ± 6.2	8.5 ± 5.4	8.7 ± 5.8	7.9 ± 5.8	T = −2.238 p = 0.034 d = 0.439	time: F = 0.670; df = 3,57; p = 0.574 treatment: F = 0.790; df = 1,19; p = 0.385 time × treatment: F = 0.683; df = 3,57; p = 0.566
	rTMS	7.6 ± 5.2	7.8 ± 4.7	8.4 ± 5.1	8.1 ± 5.1		
change in clinical global impression (1–7*)	tRNS	—	3.8 ± 0.7	4.0 ± 0.6	3.7 ± 0.7	n.a.	time: F = 1.627; df = 2,50; p = 0.207 treatment: F = 5.430; df = 1,25; p = 0.028 time × treatment: F = 0.473; df = 2,50; p = 0.648
	rTMS	—	4.1 ± 0.8	4.3 ± 0.9	4.2 ± 0.9		

*High values indicate high quality of life.

## Discussion

Treatment of chronic tinnitus with hf-tRNS is easily applicable, showed tolerable side effects, but resulted in temporal increases in tinnitus loudness in 20% of the cases leading to two drop-outs. One advantage over rTMS is that tRNS is not associated with pulsed local aversive sensations inherent to TMS stimulation. During tRNS patients report either constant slight tingling or no local sensations at all. As tRNS provides a balanced current stimulation it is considered even safer than tDCS, which induces a polarizing stimulation possibly leading to skin lesions under certain conditions^1^. Accordingly, no erythema or burning skin lesion were observed in our study. In our clinical cohort no significant effects on cognitive functioning were detected assuming tRNS of the bilateral temporal cortex as a safe procedure even if applied on a repetitive basis of 10 sessions. Decreases in performance by one standard deviation in single cases did not exceed the number of increases of performance by one standard deviation. Nonetheless, single cases with amelioration TAP-scores suggest that hf-tRNS may have potential to improve cognitive functioning under certain conditions. Even if we have no systematic or obvious deterioration in cognitive functions, further detailed investigations seem to be required.

tRNS treatment was detected to be effective in 31% of the patients who completed the treatment indicated by clinical response defined by an at least 5 point reduction in the TQ (the response rate in the intention-to-treat population was 27%). This response rate was comparable to rTMS efficacy in earlier trials of our center^26^.

A potential objection of the present study might be the missing measurement of individual hearing loss, as this is well known and broadly accepted as a central risk factor for the development of chronic tinnitus^27^. However, in predictor analyses concerning brain stimulation methods in chronic tinnitus, no impact of hearing level on clinical outcome could be detected so far^28^.

Notably, treatment response to rTMS in our study sample did not predict treatment response to tRNS. Please note, that it was not the intention of the present study to investigate the additive effects of rTMS treatment and tRNS treatment (e.g. there was no “tRNS only” control condition, and the rTMS protocols vary significantly between the participants). In addition to these arguments related to study design, potential carry-over effects cannot reliably and totally be ruled out although the authors consider them as unlikely in the view of the long (minimum 3 months) wash-out periods between the two courses of brain stimulation techniques. The exact time spans are proved in detail for each participant in a table in supplementary information. The authors suggest not to over-interpret the comparing analyses between rTMS and tRNS treatment. However, they feel that it may indeed serve as a helpful tool to estimate the potential of hf-tRNS treatment in a naturalistic setting of a variety of differential brain stimulation techniques targeting chronic tinnitus perceptions.

Only two “double responders” according to the criterion mentioned above were detected in our study cohort. This observation might favor an individualized treatment approach. For brain stimulation techniques, such an approach could be realized by utilizing single test sessions as predictors for daily treatment. Such attempts have already been suggested in rTMS applications for different stimulation targets and parameters providing innovative treatment strategies in chronic tinnitus^29^. However, it has to be stated that in the presented study we did not evaluate possible immediate effects of single-sessions of tRNS in our patients. These issues will have to be addressed in upcoming studies.

Specific changes in tinnitus unpleasantness after tRNS in contrast to rTMS might be explained by the stimulation of more medial temporal areas such as posterior insula or hippocampus as a summation effect of electric currents in border regions linking different types of tissues^4^. However, we would strongly suggest not to over-interpret this result as it might be purely attributable to multiple testing. In line with this argumentation we abstain from over-interpretation of the physical quality of life findings. No other secondary outcome over the course of the treatment was significant. In sum, for the whole group we could not demonstrate efficacy of hf-tRNS for treatment of chronic tinnitus.

A factor of uncertainty is the use of hf-tRNS (100–640 Hz) as there is evidence for efficacy of lf-tRNS in chronic tinnitus^14–16^. In sum, most tRNS studies in tinnitus used lf-tRNS and showed efficacy. One direct comparison of hf- and lf-rRNS turned out that lf-tRNS might be superior to hf-tRNS with respect to tinnitus distress^16^. Both interventions were similar effective with respect to tinnitus loudness. Hf-tRNS turned out to be more effective for pure-tone in contrast to noise-like tinnitus. Although there is evidence from steady state EEG data and auditory evoked potentials that tRNS effect to the auditory might parallel the effects in motor-cortical areas^4^, the optimal frequency spectrum for stimulation of the auditory cortex in tinnitus patients remains a matter of debate.

Another limitation of our study is the fact, that all patients had already absolved one rTMS treatment series introducing a possible selection bias. However, the fact that positive response to rTMS treatment did not predict response in the present tRNS-based study suggests different underlying mechanisms of both stimulation techniques. The authors are well aware of the fact that unspecific effects cannot be reliably ruled out. Nevertheless, the fact that all patients already participated in a neurostimulation treatment study before, makes it rather improbable that the observed effects are purely unspecific effects, as unspecific effects of rTMS and tRNS should resemble each other. In addition, an optimal design would have included a placebo group/condition. For ethical reasons, we decided to use a real-life application using a within-subjects design with the factor of different conditions.

Taken together, our pilot study provides evidence for the feasibility of high-frequency tRNS in the treatment of chronic tinnitus and demonstrated an identical low response rate compared to transcranial magnetic stimulation in a study cohort which was treated almost two years after the first neurostimulation treatment attempt with rTMS. In sum, the trial was negative as no secondary outcome measure was significant including TQ (the basis for the definition of treatment response). As the subgroup of patients who responded to hf-tRNS differed from rTMS responders, the results advocate to further focus on individualized treatment approaches.

## Supplementary information

table 2
