# Supplementary material for: Daily high-frequency transcranial random noise stimulation of bilateral temporal cortex in chronic tinnitus – a pilot study
Source: Sci Rep. 2019 Aug 22;9:12274. doi: 10.1038/s41598-019-48686-0 (PMC6706578; doi:10.1038/s41598-019-48686-0)
Supplement: Supplementary file 1 — table 2 [file 41598_2019_48686_MOESM1_ESM.docx]

**Daily high-frequency transcranial random noise stimulation**

**of bilateral temporal cortex in chronic tinnitus – a pilot study**

Peter M. Kreuzer^1,3^, Timm B. Poeppl^1,3^, Rainer Rupprecht^1^,

Veronika Vielsmeier^2,3^, Astrid Lehner^1,3^, Berthold Langguth^1,3^, Martin Schecklmann^1,3^

^1^ Department of Psychiatry and Psychotherapy, University of Regensburg, Germany

^2^ Department of Otorhinolaryngology, University of Regensburg, Regensburg, Germany

^3^ Interdisciplinary Tinnitus Center of the University of Regensburg, Regensburg, Germany

Correspondence to:

Peter M. Kreuzer, MD

Department of Psychiatry and Psychotherapy, University of Regensburg,

Universitätsstrasse 84,

93053 Regensburg, Germany.

Email: peter.kreuzer@medbo.de

Phone: +49 941 941 1256

Fax: +49 941 941 1255

**Table 2.** Descriptive statistics.

| patient number | sex (female/ male) | age (years) | tinnitus duration (months) | tinnitus laterality | tinnitus distress (tinnitus questionnaire) | hearing loss  (mean dB for all frequencies) | time of rTMS before tRNS (months) | kind of rTMS* | response to tRNS treatment | response to rTMS treatment |
| --- | --- | --- | --- | --- | --- | --- | --- | --- | --- | --- |
| 1 | m | 51 | 62 | right > left | 23 | 15,00 | 3 | 20Hz left frontal  1Hz left temporoparietal  relaxation ^1^ | yes | no |
| 2 | f | 46 | 131 | right > left | 69 | ,00 | 108 | 1Hz left temporal sham | drop-out | no |
| 3 | m | 40 | 139 | inside head | 52 | 15,00 | 29 | 20Hz left frontal  1Hz left temporoparietal  1Hz right temporoparietal | yes | no |
| 4 | m | 60 | 102 | right = left | 58 | 40,28 | 39 | 20Hz left frontal  1Hz left temporoparietal | no | no |
| 5 | m | 61 | 31 | left < right | 14 | 15,56 | 14 | 20Hz left frontal  1Hz left temporoparietal | drop-out | no |
| 6 | m | 33 | 165 | right = left | 80 | 21,67 | 31 | 20Hz left frontal  1Hz left temporoparietal  1Hz right temporoparietal | yes | yes |
| 7 | m | 65 | 188 | left | 55 | n.a. | 86 | 1Hz left temporal | no | no |
| 8 | m | 52 | 84 | left | 68 | 23,44 | 11 | 20Hz left frontal  1Hz left temporoparietal  1Hz right temporoparietal | no | no |
| 9 | m | 60 | 109 | left | 49 | 18,89 | 36 | 20Hz left frontal  1Hz left temporoparietal  1Hz right temporoparietal | yes | yes |
| 10 | m | 52 | 30 | left | 43 | 17,78 | 12 | 20Hz left frontal  1Hz left temporoparietal  1Hz right temporoparietal | no | yes |
| 11 | m | 56 | 57 | right | 19 | n.a. | 22 | 20Hz anterior cingulum  1Hz temporoparietal | no | yes |
| 12 | m | 56 | 61 | right = left | 26 | 16,67 | 49 | 20Hz left frontal  1Hz left temporal | no | no |
| 13 | m | 37 | 156 | left < right | 65 | 20,94 | 23 | 20Hz left frontal  1Hz left temporal | yes | no |
| 14 | m | 29 | 141 | right = left | 48 | 4,44 | 84 | 1Hz left temporal  L-DOPA | drop-out | no |
| 15 | m | 56 | 46 | inside head | 30 | 30,28 | 10 | 20Hz left frontal  1Hz left temporoparietal  relaxation therapy | drop-out | yes |
| 16 | m | 58 | 355 | right > left | 32 | 46,67 | 4 | 20Hz left frontal  1Hz left temporoparietal  muscle stimulation | no | yes |
| 17 | f | 60 | 16 | left | 9 | 21,39 | 4 | 20Hz left frontal  1Hz left temporoparietal  muscle stimulation | no | yes |
| 18 | m | 42 | 14 | inside head | 51 | n.a. | 5 | 20Hz left frontal  1Hz left temporoparietal  muscle stimulation | yes | no |
| 19 | f | 49 | 23 | left < right | 29 | 23,61 | 3 | 20Hz left frontal  1Hz left temporoparietal  muscle stimulation | no | yes |
| 20 | m | 54 | 56 | right = left | 49 | 42,78 | 6 | 20Hz left frontal  1Hz left temporoparietal  muscle stimulation | no | no |
| 21 | m | 58 | 40 | left | 31 | 8,61 | 32 | 20Hz left frontal  1Hz left temporal | yes | no |
| 22 | m | 60 | 113 | left | 18 | 32,50 | 6 | 20Hz left frontal  1Hz left temporoparietal  muscle stimulation | no | no |
| 23 | m | 19 | 33 | right = left | 48 | ,28 | 6 | 20Hz left frontal  1Hz left temporoparietal  muscle stimulation | no | yes |
| 24 | f | 44 | 22 | inside head | 32 | 8,61 | 3 | 20Hz left frontal  1Hz left temporoparietal  muscle stimulation | no | no |
| 25 | m | 53 | 178 | right = left | 69 | 24,72 | 23 | 20Hz anterior cingulum  1Hz temporoparietal | no | no |
| 26 | m | 38 | 77 | right = left | 57 | 13,61 | 4 | 20Hz left frontal  1Hz left temporoparietal  muscle stimulation | no | no |
| 27 | m | 36 | 115 | right > left | 58 | n.a. | 6 | 20Hz left frontal  1Hz left temporoparietal | no | no |
| 28 | m | 57 | 183 | inside head | 56 | 30,00 | 6 | 20Hz left frontal  1Hz left temporoparietal  muscle stimulation | no | no |
| 29 | m | 49 | 29 | right = left | 74 | 29,29 | 8 | 20Hz left frontal  1Hz left temporoparietal  muscle stimulation | no | no |
| 30 | m | 47 | 123 | right = left | 61 | 1,11 | 6 | 20Hz left frontal  1Hz left temporoparietal  muscle stimulation | yes | no |

rTMS = repetitive transcranial magnetic stimulation. tRNS=transcranial random noise stimulation.

*Each patients received two weeks of daily (Monday-Friday) rTMS treatment.

rTMS = repetitive transcranial magnetic stimulation. tRNS=transcranial random noise stimulation.

*Each patients received two weeks of daily (Monday-Friday) rTMS treatment
